# Interpreting household survey data intended to measure insecticide-treated bednet coverage: results from two surveys in Eritrea

**DOI:** 10.1186/1475-2875-5-36

**Published:** 2006-05-05

**Authors:** Thomas P Eisele, Kate Macintyre, Josh Yukich, Tewolde Ghebremeskel

**Affiliations:** 1 Department of International Health and Development, Tulane University School of Public Health and Tropical Medicine, 1440 Canal St., Suite 2200, New Orleans LA 70115, USA; 2 National Malaria Control Programme, Ministry of Health, Eritrea

## Abstract

**Background:**

As efforts are currently underway to roll-out insecticide-treated bednets (ITNs) to populations within malarious areas in Africa, there is an unprecedented need for data to measure the effectiveness of such programmes in terms of population coverage. This paper examines methodological issues to using household surveys to measure core Roll Back Malaria coverage indicators of ITN possession and use.

**Methods:**

ITN coverage estimates within Anseba and Gash Barka Provinces from the 2002 Eritrean Demographic and Health Survey, implemented just prior to a large-scale ITN distribution programme, are compared to estimates from the same area from a sub-national Bednet Survey implemented 18 months later in 2003 after the roll-out of the ITN programme.

**Results:**

Measures of bednet possession were dramatically higher in 2003 compared to 2002. In 2003, 82.2% (95% confidence interval (CI) 77.4–87.0) of households in Anseba and Gash Barka possessed at least one ITN. RBM coverage indicators for ITN use were also dramatically higher in 2003 as compared to 2002, with 76.1% (95% CI 69.9–82.2) of children under five years old and 52.4% (95% CI 38.2–66.6) of pregnant women sleeping under ITNs. The ITN distribution programme resulted in a gross increase in ITN use among children and pregnant women of 68.3% and 48% respectively.

**Conclusion:**

Eritrea has exceeded the Abuja targets of 60% coverage for ITN household possession and use among children under five years old within two malarious provinces. Results point to several important potential sources of bias that must be considered when interpreting data for ITN coverage over time, including: disparate survey universes and target populations that may include non-malarious areas; poor date recall of bednet procurement and treatment; and differences in timing of surveys with respect to malaria season.

## Background

Insecticide-treated bednets (ITNs) have been shown to significantly reduce malaria transmission and concomitant malaria related morbidity and all-cause child mortality across a range of transmission settings, even with sustained use [1-6]. ITN use during pregnancy has also been shown to significantly reduce the prevalence of low birth weight deliveries and malaria related morbidity among pregnant women [7].

Efforts are currently underway to roll-out ITNs to populations across sub-Saharan Africa with increased funding from the Global Fund to Fight AIDS, Tuberculosis and Malaria, the World Bank Global Strategy and Booster Program, the President's Malaria Initiative (PMI) and the Bill and Melinda Gates Foundation among others. Accordingly, there is an unprecedented need for data to measure the effectiveness of ITN distribution programmes in terms of population coverage [8]. The Abuja Summit set targets for ITN coverage by 2005 of 60% among vulnerable populations at risk for malaria [9], while the World Health Assembly and the PMI have recently set loftier targets of 80% and 85%, respectively, for coverage among vulnerable populations by the end of the decade [10,11]. To measure progress towards such targets, the Roll Back Malaria (RBM) partnership has established a set of core indicators to measure ITN coverage at the household and individual level [12]. Data for these indicators will rely almost exclusively on probability household surveys such as the Demographic and Health Survey (DHS), UNICEF's Multiple Indicator Cluster Survey (MICS), the Malaria Indicator Survey, as well as other local surveys being implemented within malarious areas. Ideally, data on ITN coverage will be collected in a way that allows for longitudinal assessment of trends over time. As such, there is a need for surveys to be reliable and comparable over time and between countries.

Two household surveys implemented 18 months apart that measured ITN coverage within two malarious zobas (zones) of Eritrea are compared. Issues of interpreting cross-sectional survey data for measuring core RBM coverage indicators of ITN possession and use among children less than five years old and pregnant women are explored. Additionally, we demonstrate how an evaluation of a national programme is possible using these data from two cross sectional surveys, and present gross programme effects useful for managers and public health strategists as they focus on moving forward with the global agenda to protect populations most at risk of malaria.

## Methods

### Study area

Eritrea, a country of 3.5 million, is divided into 6 administrative provinces or *zobas*. Malaria transmission is seasonal, concentrated primarily in the low-lying areas of three zobas, Anseba, Gash-Barka and Debub from August through October [13]. While transmission is moderate with population estimates of malaria parasitemia ranging from 0.4% to 6.5% [13], malaria has historically represented a significant public health problem, accounting for 28% of all hospital admissions and 30% of all outpatient morbidity [13,14].

The Eritrean National Malaria Control Programme (NMCP) began a scaled-up ITN distribution and re-impregnation programme at the end of 2002, focused primarily within malarious zobas. The programme was designed to provide each household with two ITNs free of charge, while providing insecticide treatment annually for all existing bednets. As part of this programme, ITNs were also distributed free of charge to all pregnant women in the programme area through antenatal clinics. It is estimated that by the end of the 2003 over 500,000 ITNs had been distributed within the programme area.

**Figure 1:**
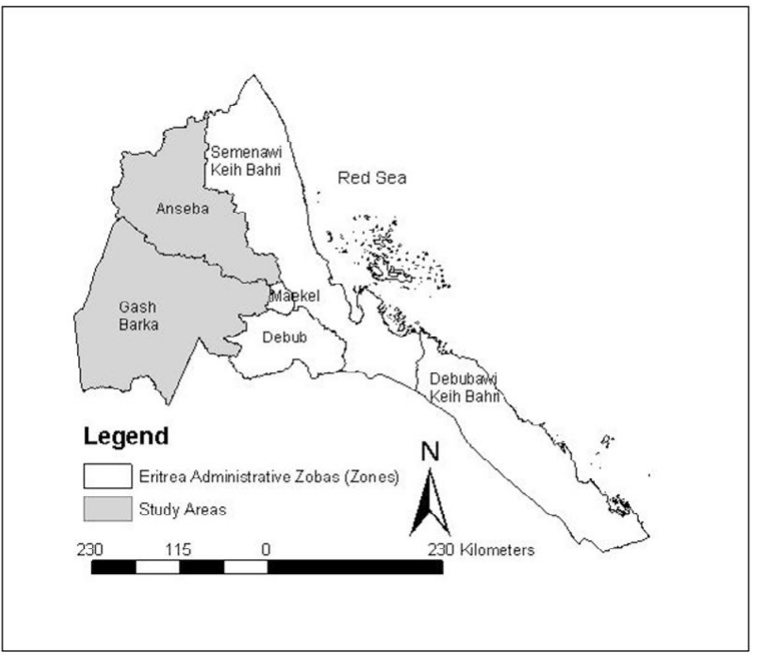
Map of Eritrea showing the two zobas used in comparing the Eritrean Demographic and Health Survey (2002) and the Bednet Survey (2003): Anseba and Gash Barka.

The scope of this comparison between the 2002 Eritrea DHS (EDHS) and the 2003 Bednet Survey has been limited to Anseba and Gash Barka because the sampling frames used in these two survey domains were similar (Figure 1). While nearly all sub-zobas of Debub were eligible for inclusion in the EDHS, 6 sub-zobas of Debub were excluded from the sampling frame in the Bednet Survey for security and logistical issues [15].

### Eritrean demographic and health survey (2002)

The EDHS was carried out in the dry season just prior to the roll-out of the NMCP ITN distribution programme from April through June 2002 in all zobas of Eritrea. Details of the sampling design are described elsewhere [16]. Briefly, a two-stage cluster design, with first stage probability proportional to estimated cluster size (PPES), was used to obtain a nationally-representative probability sample of households. Within selected primary sampling units (PSUs), households were selected at second stage using systematic sampling from a complete enumeration of households. In total, 9,389 households were successfully interviewed in Eritrea in the EDHS, of which 3,138 were from Anseba and Gash Barka. Information at the household level, including the number of bednets, was obtained from the head of household. Information on bednet use among women and their children was obtained from interviews with all women of reproductive age within selected households (Anseba n = 1,450 women; Gash Barka n = 1,446 women).

### Bednet survey (2003)

The Bednet Survey was carried out during the rainy season of September and October 2003, approximately one year after the roll-out of the NMCP ITN distribution programme. As recommended by the RBM guidelines for measuring core coverage indicators for ITN possession and use, the Bednet Survey was intentionally conducted during the rains to capture the period when members of the respective household were most likely to be *using *the nets [12]. Details of the sampling strategy have been described elsewhere [15]. Briefly, a modified two-stage cluster design, with PPES first stage, was used to obtain a probability sample of households within Anseba, Gash-Barka and Debub. The three zobas were treated as separate survey domains and sampled independently using equal allocation. Second stage selection was based on a modified segmentation design where PSUs were divided into segments of equal size of approximately 26 households, from which one segment per PSU was selected and all households included in the sample [17]. The survey yielded a total sample size for Anseba and Gash Barka of 1,559 households from 60 villages. All information for this survey, including bednet use among women and children, was obtained from an interview with the head of household. Information on all children within the household was obtained, regardless of whether their mother was a resident of the household. It should be noted that data for socio-economic status of the household was not collected as part of the Bednet Survey as it was not hypothesized to be a determinant of household ITN possession or use in this area of Eritrea because of the extremely low variability of wealth in this target population.

### Data analysis

SAS 8.2 was used for all data management and statistical analysis. Data from the EDHS were weighted based on relative strata size and corrections to the relative size of PSUs within strata. Data from the Bednet Survey were weighted based on relative strata size only. To account for the effect of inter-cluster correlations due to the two stage cluster designs, all standard errors were estimated using the Taylor expansion method [18].

Design effect is used to compare the EDHS and Bednet Survey in terms of the relative loss of precision as a result of each cluster sampling method, calculated as the ratio between the standard error of each sampling design, using empirically estimated standard errors, and the standard error that would result had simple random sampling been used (SE_2-stage design_/SE_simple random sampling_). Relative standard errors are used to compare the relative precision for each point estimate between the EDHS and the Bednet Survey, calculated as the point estimate divided by its standard error.

All select demographic variables were ascertained from household respondents and are presented at the household level. A bednet is defined as any mosquito net used for sleeping, treated or untreated. To remain consistent with the RBM coverage indicators, an ITN is defined as any bednet that was either procured or dipped in insecticide within the previous 12 months (≤ 11 months) [12]. All bednets procured within the previous 12 months were assumed to be treated. This was rationalized as almost all new bednets distributed in the area are either pretreated or permanently treated with insecticide. It should be noted that the EDHS did not collect information on bednet treatment at the household level (only at the individual level for children and women of reproductive age), thus the proportion of households with at least one ITN could not be calculated directly. The number of months since bednet procurement and insecticide treatment were smoothed due to significant date heaping at 12 months. For all months since procurement and treatment equal to 12, half were randomly assigned to 11. This procedure was followed because for all respondents who answered "one year" to the length of time since bednet procurement or treatment, it is deemed equally likely the exact number of months to these events would have actually been greater than or less than 12 months.

Estimates for the proportion of children under five years old and pregnant women who slept under an ITN the previous night for the EDHS and Bednet Survey were constructed based on the recommended RBM coverage indicators for these target populations [12].

### Ethical procedures

Ethical clearance for the study protocol was obtained from the institutional review board of Tulane University and the Ministry of Health in Eritrea.

## Results

There was little difference between the EDHS and the Bednet Survey in terms of their relative precision resulting from their different cluster sampling designs, as measured by design effect (Table 1). The design effects of the two-stage cluster design used in the EDHS for select demographic and ITN coverage variables within Anseba and Gash-Barka ranged from 1.27 to 4.46, with a mean of 2.46, while the design effects of the modified two-stage cluster design with segmentation used by the Bednet Survey for these demographic variables ranged from 1.17 to 4.69, with a mean of 2.59. The relative standard errors for these demographic and ITN coverage variables ranged from 1.8%–37.5% for the EDHS and 2.3%–20.2% for the Bednet Survey.

**Table 1:** Survey parameters of select demographic and ITN coverage indicators for the Eritrean Demographic and Health Survey (2002) and the Bednet Survey (2003)

**Indicator**	**Survey**	**Sample size**	**Proportion (unweighted)**	**Proportion (weighted)**	**Standard error**	**Design effect**	Relative standard error (%)*
Proportion households with at least 1 child ≤ 5 years old	EDHS	3,138	0.5102	0.5063	0.0114	1.27	2.2
	Bednet Survey	1,559	0.5710	0.5745	0.0153	1.17	2.6
Proportion households with ≥ 4 members	EDHS	3,138	0.6673	0.6598	0.0117	1.39	1.8
	Bednet Survey	1,559	0.6459	0.6469	0.0152	1.25	2.3
Proportion households respondent Muslim^†^	DHS	2,832	0.6207	0.6199	0.0381	4.18	6.2
	Bednet Survey	1,559	0.6421	0.6385	0.0570	4.69	8.9
Proportion household respondents Tigrinya	DHS	3,138	0.2954	0.3059	0.0364	4.46	11.9
	Bednet Survey	1,559	0.2348	0.2433	0.0491	4.57	20.2

Proportion households with at least 1 bednet	EDHS	3,138	0.4783	0.4982	0.0246	2.76	4.9
	Bednet Survey	1,559	0.9268	0.9265	0.0153	2.31	1.6
Proportion households with at least 1 ITN^§^	EDHS^‡^	-	-	-	-	-	-
	Bednet Survey	1,559	0.8287	0.8217	0.0243	2.55	3.0
Proportion children under 5 who slept under ITN previous night^§^	EDHS	2,089	0.0732	0.0781	0.0107	1.88	13.7
	Bednet Survey	1,488	0.7655	0.7605	0.0314	2.86	4.1
Proportion pregnant women who slept under ITN previous night^§^	EDHS	279	0.0466	0.0435	0.0163	1.29	37.5
	Bednet Survey	78	0.5256	0.5238	0.0723	1.27	13.8

*Standard error/proportion (weighted)

^†^Proportion Muslim for EDHS based on women of reproductive age; proportion Muslim for Bednet Survey based on household respondent.

^‡^No data available on bednet treatment status at household level from EDHS

^§^Data smoothed for date heaping at 12 months for defining ITN.

**Figure 2:**
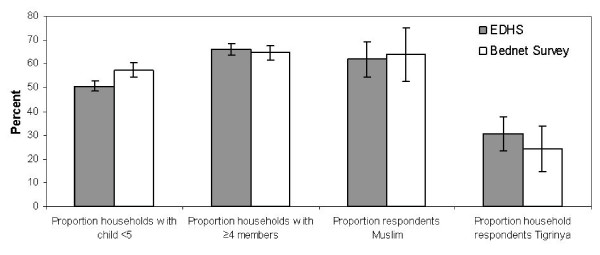
Comparison between select demographic indicators measured by the Eritrean Demographic and Health Survey (EDHS) (2002) and the Bednet Survey (Bednet Survey) (2003), Anseba and Gash Barka. Sample size (n) for all indicators from EDHS except proportion Muslim is equal to households (3,138); sample size (n) for all indicators from Bednet Survey is equal to households (1,559). Proportion Muslim for EDHS based on women of reproductive age (n = 2,832) while proportion Muslim for Bednet Survey based on household respondents.

Results of select household demographic variables were compared to assess the overall comparability of the two surveys (Figure 2). All such variables were very similar between the EDHS and the Bednet Survey. The only variable with 95% confidence intervals that did not overlap was the proportion of households with at least one of the members a child under five years old, which was 50.6% (95% confidence interval (CI) 48.4–52.8) for the EDHS and 57.5% (95% CI 54.4–60.4) for the Bednet Survey.

**Figure 3:**
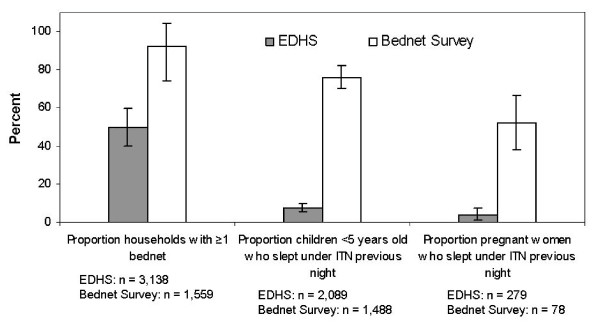
Comparison between ITN coverage indicators measured by the Eritrean Demographic and Health Survey (2002) and the Bednet Survey (2003), Anseba and Gash Barka.

Measures of bednet possession were dramatically higher on the Bednet Survey (2003) compared to the EDHS (2002) within Anseba and Gash-Barka (Figure 3). The proportion of households with at least one bednet estimated by the Bednet Survey was nearly double (92.7%; 95% CI 89.7–95.6) that estimated by the EDHS (49.8%; 95% CI 45.0–54.6). Accordingly, the mean number of bednets per household estimated by the Bednet Survey (2.048; 95% CI 1.890–2.205) was more than double that of the EDHS (0.847; 95% CI 0.738–0.957), while the proportion of households with two or more bednets increased from 23.19% on the EDHS (95% CI 19.23–27.14) to 65.41% (59.94–70.88) on the Bednet Survey.

While the RBM coverage indicator for household ITN possession was not measured on the EDHS, over three-quarters (82.2%; 95% CI 77.4–87.0) of households were estimated to possess at least one ITN from the Bednet Survey. Additionally, over half (55.16%; 95% CI 49.56–60.76) of the households sampled on the Bednet Survey owned at least two ITNs, with the mean number of ITNs per household equal to 1.752 (95% CI 1.593–1.911).

RBM coverage indicators for ITN use among children and pregnant women within Anseba and Gash-Barka were dramatically higher on the Bednet Survey in 2003 as compared to the EDHS in 2002 (Figure 3). Over three-quarters (76.1%; 95% CI 69.9–82.2) of children under five years old were estimated to have slept under an ITN the previous night on the Bednet Survey, as compared to only 7.8% (95% CI 5.7–9.9) on the EDHS, resulting in gross programme effect of 68.3% [76.1% (Bednet Survey) – 7.8% (EDHS)]. Over half (52.4%; 95% CI 38.2–66.6) of pregnant women were estimated to have slept under an ITN the previous night on the Bednet Survey, as compared to only 4.4% (95% CI 1.2–7.5) on the EDHS, resulting in a gross programme effect of 48.0% [52.4% (Bednet Survey) – 4.4% (EDHS)].

## Discussion

This paper examines the results of two household surveys on ITN coverage indicators for household possession and use among children under five years old and pregnant women within two malarious areas of Eritrea pre and post roll-out of a full-coverage ITN distribution programme.

Based on results from the 2003 Bednet Survey, Eritrea has exceeded the Abuja targets of 60% coverage for ITN household possession (82.2%) and use among children under five years old (76.1%) within two malarious provinces. Use among pregnant women in 2003 (52.4%) increased dramatically from 2002 (5.2%) and approaches the Abuja target of 60%. However, the World Health Assembly passed a resolution in 2005 urging member states to increase coverage of such malaria control interventions among vulnerable populations to 80% by the end of the decade, thus there is still work to be done [10].

Household selection using segmentation at second stage appears to be a suitable alternative to the gold-standard two-stage cluster design employed by the EDHS in terms of design effect and relative precision for obtaining data for ITN coverage indicators. This may be important when survey resources are scarce, as the field implementation of the Bednet Survey was conducted for approximately US$ 50,000 for all three zobas (US$ 21 per house for total sample size of 2,340 for three zobas), compared to approximately US$ 500,000 for the EDHS (US$ 53 per house for sample size of 9,389), resulting in a savings in cost per house sampled of $US 32. However, the latter covered the entire country and included a much broader spectrum of health parameters.

Unfortunately, no data on the timing of bednet procurement, bednet type or treatment status was collected at the household level on the 2002 EDHS. This precluded the calculation of the recommended RBM coverage indicator for household ITN possession. However, of the children that slept under a bednet on the 2002 EDHS, 49.9% of the bednets had either been treated or procured within the last 12 months. Thus using this figure in conjunction with the proportion of households on the EDHS that possessed a bednet (49.8%), it can be extrapolated that 24.9% of households on the EDHS likely possessed at least one ITN in 2002. It is recognized this may represent an overestimate of household ITN possession as a bednet within a house with a child sleeping under it may be more likely to have been treated compared to a bednet within a household without a child sleeping under it. Using this estimate from 2002, the gross effect of the NMCP distribution programme is estimated to have contributed to a 57.3% increase in household ITN possession between pre and post roll-out [82.2% (Bednet Survey) – 24.9% (EDHS)].

These results demonstrate that the NMCP ITN distribution programme was effective in increasing ITN household possession and use among children under five and pregnant women in Anseba and Gash Barka. However, better estimates of the net programme effect are required to determine the exact level of the ITN coverage increase that can be attributed directly to the distribution programme. Unfortunately, multilevel analyses that account for non-programme effects on the outcomes of ITN coverage between the two surveys were not possible due to the limitations of the cross-sectional pre-post evaluation design and lack of a suitable control group due to the nature of the full coverage programme. While the two surveys were relatively comparable over time, results point to several important factors that must be considered when interpreting household survey data for assessing progress towards global targets.

It is expected that at least some of the gross programme effect observed between these surveys can be attributed to the fact that individuals would have been more likely to use ITNs during the Bednet Survey which was implemented during the rainy season when mosquito nuisance would have been more common as compared to the EDHS that was implemented in the dry season [19-22]. This season effect is at least partially illustrated by the fact that while almost twice as many houses were found to own a bednet on the Bednet Survey compared to the EDHS (92.7% compared to 49.8%, respectively), ITN use among children was nearly tenfold higher on the Bednet Survey (76.0%) compared to the EDHS (7.8%). This suggests that among houses with bednets, their members are more likely to use them during the rainy season. It is therefore recommended that household survey designs account for the effect of season on ITN use.

As global targets are intended to be measured among those at risk for malaria, inclusion of populations not at risk for malaria, such as those within heavily urban areas, highland areas or within extremely arid regions, in the numerator and denominator will likely yield biased estimates of RBM ITN coverage indicators [19]. The two surveys presented here cover the same geographical areas and target population and thus point estimates are relatively comparable over time. Both urban and rural populations were included in our analysis because Anseba and Gash Barka are overwhelmingly rural (> 80% within rural areas on EDHS). However, when interpreting national-level estimates of ITN coverage indicators, we recommend estimates be disaggregated by urban and rural strata at a minimum, and if possible by highland (> 300 meters) versus lowland areas where applicable. The importance of disaggregating between urban and rural populations is illustrated by the finding that in many instances, urban populations, which are typically at a much lower risk for malaria, routinely have higher bednet coverage, likely due to better access to ITN outlets and socioeconomic factors [22].

**Figure 4:**
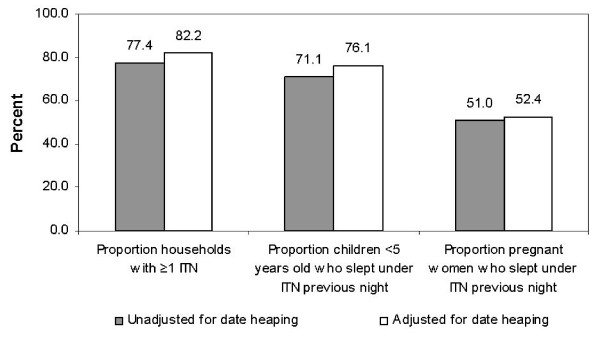
ITN coverage point estimates adjusted and unadjusted for date heaping at 12 months: Bednet Survey (2003), Anseba and Gash Barka. For adjusted estimates, for all months since procurement and treatment equal to 12, half were randomly assigned to 11.

Recall bias of respondents within both the EDHS and Bednet Survey resulted in substantial date heaping at 12 months, or "one year", for the time since a bednet was procured and treated. This is best illustrated by the extent of date heaping on the Bednet Survey where of the 1439 households with a bednet that reported the number of months since it was procured, nearly half (47.5%) reported "one year" or 12 months, with no households stating 11 or 13 months. Accordingly, date heaping at 12 months had a significant effect on differentiating between a bednet and an ITN as defined by the RBM indicators [12]. Differences in resulting point estimates adjusted and unadjusted for date heaping are most dramatically illustrated by results from the Bednet Survey (Figure 4). These results demonstrate that ignoring date heaping at 12 months may lead to underestimates of ITN coverage, which may prove significant as countries attempt to reach the Abuja targets set at 60%.

## Authors' contributions

TE helped conceive this research, performed the statistical analysis and led the drafting of this manuscript. KM conceived this research and helped with drafting of this manuscript; JY helped with statistical analysis and drafting of the manuscript; TG helped conceive this research and reviewed the manuscript. All authors read and approved the final manuscript.

## Financial support

The work presented here was funded through two projects: 1) the American Schools of Public Health/Centers for Disease Control and Prevention cooperative agreement (S1942-21/21), Enhancing National Malaria Control in Eritrea; and 2) the Environmental Health Project Contract # HRN-1-00-99-00011-00. Support also came through the Ministry of Health, Eritrea and Tulane University.
